# Donor-derived cell-free DNA monitoring for early diagnosis of antibody-mediated rejection after kidney transplantation: a randomized trial

**DOI:** 10.1093/ndt/gfae282

**Published:** 2024-11-29

**Authors:** Aylin Akifova, Klemens Budde, Kerstin Amann, Maike Buettner-Herold, Mira Choi, Michael Oellerich, Julia Beck, Kirsten Bornemann-Kolatzki, Ekkehard Schütz, Friederike Bachmann, Fabian Halleck, Ellen von Hoerschelmann, Nadine Koch, Eva Schrezenmeier, Evelyn Seelow, Johannes Waiser, Bianca Zukunft, Kai-Uwe Eckardt, Jan Halbritter, Ralph Kettritz, Covadonga López Del Moral, Nils Lachmann, Diana Stauch, Matthias Niemann, Danilo Schmidt, Philip F Halloran, Bilgin Osmanodja

**Affiliations:** Department of Nephrology and Intensive Care, Charité – Universitätsmedizin Berlin, Berlin, Germany; Department of Nephrology and Intensive Care, Charité – Universitätsmedizin Berlin, Berlin, Germany; Department of Nephropathology, Institute of Pathology, University Hospital Erlangen, Friedrich-Alexander University Erlangen-Nuremberg, Erlangen, Germany; Department of Nephropathology, Institute of Pathology, University Hospital Erlangen, Friedrich-Alexander University Erlangen-Nuremberg, Erlangen, Germany; Department of Nephrology and Intensive Care, Charité – Universitätsmedizin Berlin, Berlin, Germany; Department of Clinical Pharmacology, University Medical Center Göttingen, Göttingen, Germany; Chronix Biomedical GmbH, Göttingen, Germany; Chronix Biomedical GmbH, Göttingen, Germany; Chronix Biomedical GmbH, Göttingen, Germany; Department of Nephrology and Intensive Care, Charité – Universitätsmedizin Berlin, Berlin, Germany; Department of Nephrology and Intensive Care, Charité – Universitätsmedizin Berlin, Berlin, Germany; Department of Nephrology and Intensive Care, Charité – Universitätsmedizin Berlin, Berlin, Germany; Department of Nephrology and Intensive Care, Charité – Universitätsmedizin Berlin, Berlin, Germany; Department of Nephrology and Intensive Care, Charité – Universitätsmedizin Berlin, Berlin, Germany; Department of Nephrology and Intensive Care, Charité – Universitätsmedizin Berlin, Berlin, Germany; Department of Nephrology and Intensive Care, Charité – Universitätsmedizin Berlin, Berlin, Germany; Department of Nephrology and Intensive Care, Charité – Universitätsmedizin Berlin, Berlin, Germany; Department of Nephrology and Intensive Care, Charité – Universitätsmedizin Berlin, Berlin, Germany; Department of Nephrology and Intensive Care, Charité – Universitätsmedizin Berlin, Berlin, Germany; Department of Nephrology and Intensive Care, Charité – Universitätsmedizin Berlin, Berlin, Germany; Department of Nephrology and Intensive Care, Charité – Universitätsmedizin Berlin, Berlin, Germany; Valdecilla Biomedical Research Institute (IDIVAL), Santander, Spain; Centre for Tumor Medicine, Histocompatibility & Immunogenetics Laboratory, Charité – Universitätsmedizin Berlin, Berlin, Germany; Centre for Tumor Medicine, Histocompatibility & Immunogenetics Laboratory, Charité – Universitätsmedizin Berlin, Berlin, Germany; PIRCHE AG, Berlin, Germany; Business Division IT, Department of Research and Teaching, Charité – Universitätsmedizin Berlin, Berlin, Germany; University of Alberta, Edmonton, AB, Canada; Department of Nephrology and Intensive Care, Charité – Universitätsmedizin Berlin, Berlin, Germany

**Keywords:** biomarkers, cell-free nucleic acids, graft rejection, kidney transplantation, randomized controlled trial

## Abstract

**Background:**

Donor-derived cell-free DNA (dd-cfDNA) shows good diagnostic performance for the detection of antibody-mediated rejection (AMR) in kidney transplant recipients (KTR). However, the clinical benefits of dd-cfDNA monitoring need to be established. Early diagnosis of AMR at potentially reversible stages may be increasingly important due to emerging treatment options for AMR. We hypothesized that monitoring dd-cfDNA in KTR with *de novo* donor-specific anti-HLA antibodies (dnDSA) and performing kidney biopsy in case of increased dd-cfDNA may reduce time to AMR diagnosis in comparison with clinical indication biopsy.

**Methods:**

In this diagnostic, single-center, open-label, randomized clinical trial, we assigned 40 KTR with prevalent dnDSA and estimated glomerular filtration rate ≥20 mL/min/1.73 m^2^, but without previous biopsy-proven AMR, to either dd-cfDNA-guided biopsy (intervention group) or clinician-guided biopsy (control group) over a 12-month period. In both groups, dd-cfDNA was assessed at inclusion and 1, 3, 6, 9 and 12 months. In the intervention group, dd-cfDNA >50 copies/mL indicated a biopsy. Biopsies for clinical indication could be performed at any point during the study period in both groups. A protocol biopsy was scheduled after 12 months for patients without dd-cfDNA-guided biopsy or clinical indication biopsy until study completion. The primary endpoint was time from study inclusion to diagnosis of active or chronic active AMR.

**Results:**

Thirty-nine of 40 patients had functioning grafts at study completion. From these, 26 patients underwent biopsy, 13 in each group. AMR was diagnosed earlier in the intervention group than in the control group [median 2.8 months, interquartile range (IQR) 1.7–5.3 vs median 14.5 months, IQR 13.3–16.7, *P* = .003]. Longitudinal dd-cfDNA monitoring had 77% positive predictive value and 85% negative predictive value for AMR.

**Conclusions:**

Dd-cfDNA-guided biopsy in KTR with prevalent dnDSA can reduce the time to AMR diagnosis and hereby expedite therapy initiation.

**Trial registration:**

ClinicalTrials.gov, NCT04897438.

## Graphical Abstract

**Graphical Abstract:**
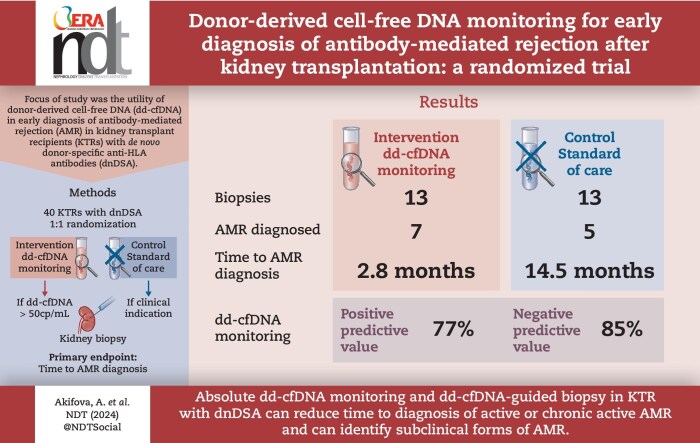

KEY LEARNING POINTS **What was known:**

- Antibody-mediated rejection (AMR) leads to graft loss in kidney transplant recipients with *de novo* donor-specific anti-HLA antibodies (dnDSA).
- Donor-derived cell-free DNA (dd-cfDNA) detects AMR more accurately than routine biomarkers such as creatinine or urine albumin.
- It is unknown how dd-cfDNA testing should be integrated into diagnostic pathways to improve clinically meaningful outcomes.

**This study adds:**

- Dd-cfDNA-guided biopsy enables earlier diagnosis of AMR in patients with dnDSA compared with clinician-guided biopsy.
- Dd-cfDNA-guided biopsy identifies subclinical AMR in stable patients with dnDSA.

**Potential impact:**

- Early identification of patients with AMR at potentially reversible stages can lead to improved outcomes, especially due to emerging treatment options for AMR.
- Dd-cfDNA can close the diagnostic gap between dnDSA monitoring and diagnosis of AMR.

## INTRODUCTION

Antibody-mediated rejection (AMR) is among the most frequent causes for graft loss following kidney transplantation [1, 2]. Non-adherence to immunosuppression, iatrogenic underexposure, previous rejection episodes and donor-specific anti-HLA antibodies (DSA) are established risk factors contributing to the complex alloimmune response that ultimately leads to AMR [3].

Both clinically significant and subclinical forms of AMR are associated with poor prognosis and are resistant to previously suggested treatment regimens [4–7]. The current diagnostic approach cannot capture the clinically silent progression of AMR, which provides a potential explanation for this therapeutic failure. Routine clinical parameters, such as creatinine and urine albumin–creatinine ratio (uACR), cannot distinguish between injury patterns, making allograft biopsy the best available, yet imperfect gold standard to verify rejection. Besides known limitations such as interobserver variability, error-prone sampling and the nonspecificity of histological lesions, the uncertainty about the appropriate biopsy timing is also of critical relevance [8–10]. In particular, AMR detected in indication biopsies frequently shows signs of chronic rejection and irreversible fibrotic changes, making therapeutic interventions less likely to be efficacious [11–13]. Conversely, performing protocol biopsies in subclinical kidney transplant recipients (KTR) with DSA confirms AMR in about half of the cases, which makes it an imprecise and invasive alternative for early diagnosis that can still miss rejection on the molecular level [14–16].

As novel therapeutic candidates, such as the CD38 monoclonal antibody felzartamab, showed very promising early results in phase 2 trials, redefining strategies for timely detection of AMR is essential to create an optimal intervention window and obtain more favorable outcomes [17].

Testing for DSA is the fundamental diagnostic step in identifying KTR at risk for AMR. While screening for preexisting DSA with the crossmatch test prior to transplantation has become a standard approach, there is no uniformly accepted algorithm for *de novo* DSA (dnDSA) monitoring post-transplant [18]. Since not all patients develop AMR after dnDSA occurrence, it is assumed that not all dnDSA are equally harmful. Despite some attempts to link certain antibody properties, such as mean fluorescent intensity (MFI), complement-binding capacity or immunoglobulin G (IgG) subclasses to higher risk for AMR and more adverse outcomes, they are unable to decipher dnDSA pathogenicity, and thus remain of indefinite diagnostic value for routine monitoring [19–22]. Moreover, dnDSA can undergo fluctuations, persist or disappear over time, which does not necessarily correlate with the clinical course [23, 24]. Thus, an additional tool that detects the onset of antibody-mediated damage could help to overcome these limitations and close the current diagnostic gap through longitudinal injury assessment [18].

Donor-derived cell-free DNA (dd-cfDNA) is an emerging biomarker in transplantation that non-invasively detects graft pathologies with increased cellular damage, such as allograft rejection [25, 26]. Its short half-life and injury-dependent release lead to superior diagnostic performance compared with the current standard-of-care parameters, especially for distinguishing AMR from no rejection [27–31]. The successful validation of different methods and their increasing availability facilitate the integration of dd-cfDNA testing into clinical practice, but evidence-based implementation strategies are lacking [32–35].

Instead of screening for AMR in large heterogeneous cohorts, we aimed to examine the potential clinical benefit of dd-cfDNA in a more concise cohort of KTR with prevalent dnDSA, but without previous biopsy after dnDSA occurrence, who have a high pre-test probability for AMR. We conducted an investigator-initiated, diagnostic, single-center, open-label, 1:1 randomized clinical trial to evaluate the influence of dd-cfDNA-guided kidney allograft biopsy (intervention group) in comparison with clinician-guided biopsy (control group) on the time to diagnosis of biopsy-proven AMR. Thus, we hypothesized that longitudinal dd-cfDNA monitoring combined with dd-cfDNA-guided biopsy can reduce the time to AMR diagnosis compared with clinician-guided biopsy in this specific group of patients.

## MATERIALS AND METHODS

### Trial design

The trial was conducted from 17 June 2021, to 18 July 2023, at the Department of Nephrology and Medical Intensive Care, Charité Universitätsmedizin Berlin (Germany). It was registered at ClinicalTrials.gov (NCT04897438) on 18 May 2021 and approved by the ethics committee of Charité (EA4/045/021; 30 March 2021). This study was conducted in accordance with the principles of the Declaration of Helsinki and the Declaration of Istanbul as outlined in the ‘Declaration of Istanbul on Organ Trafficking and Transplant Tourism’. All study participants provided written informed consent prior to study entry.

### Participants

**Figure 1:**
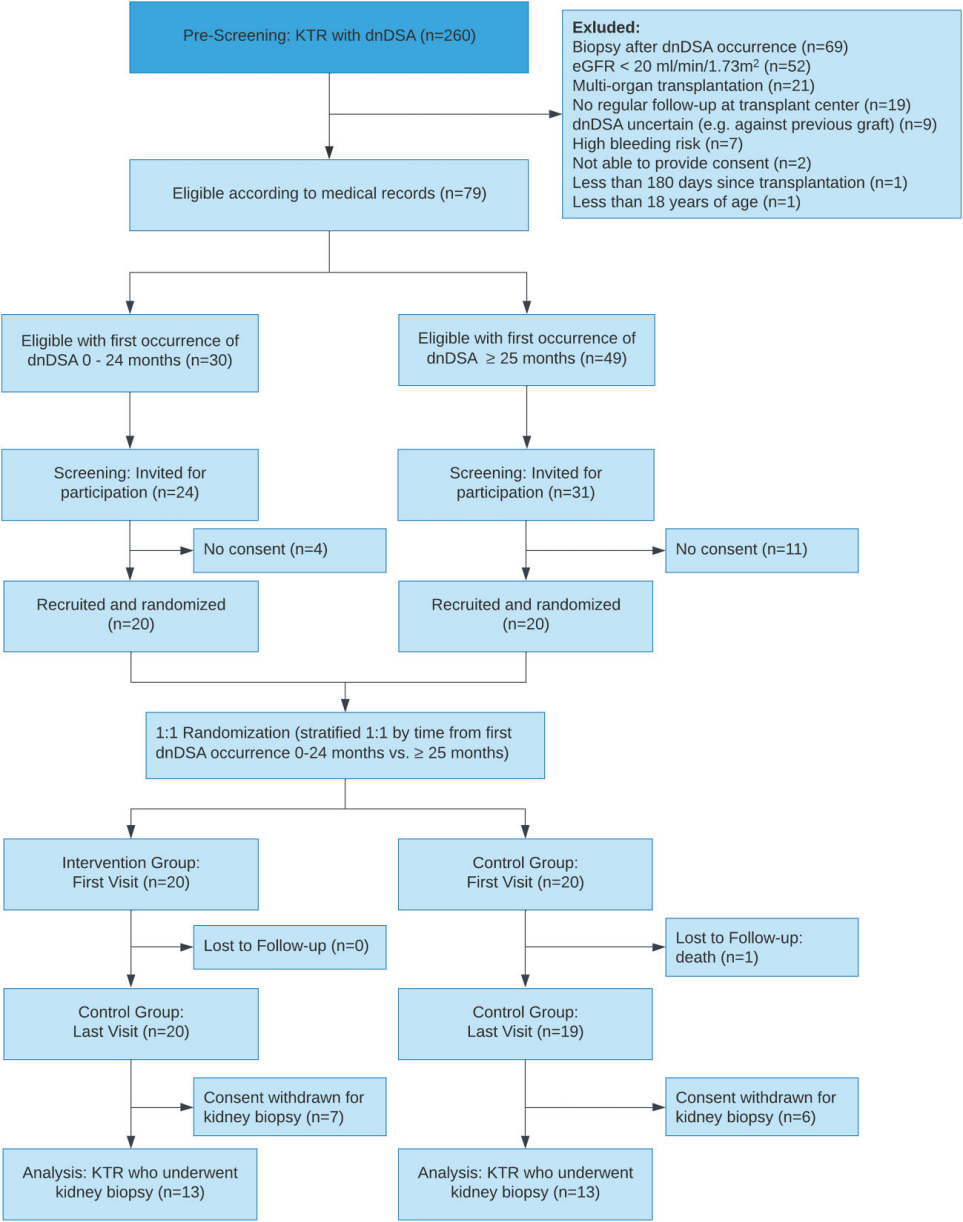
CONSORT diagram. We identified 260 KTR with prevalent dnDSA (MFI >1000) detected by annual screening out of which 79 fulfilled the eligibility criteria. From these, 55 were invited to participate during the recruitment period from 17 June 2021, until 4 July 2022, and 40 provided consent and were randomized. Since 1 patient died, and 13 withdrew consent for biopsy, 26 patients were included in the final analysis.

Adult KTR >180 days after kidney transplantation, with dnDSA against the most recent kidney transplant and an estimated glomerular filtration rate (eGFR) ≥20 mL/min/1.73 m^2^ were eligible to participate. Key exclusion criteria were multi-organ transplantations, kidney allograft biopsy after the detection of dnDSA, and increased bleeding risk. The complete inclusion and exclusion criteria are shown in Table 1 and the screening process is outlined in Fig. 1.

**Table 1:** Inclusion and exclusion criteria.

Inclusion criteria:
● Patients after kidney transplantation
● Functioning kidney transplant, at least 180 days after last transplantation
● Patients 18 years or older
● Patients provided written informed consent
● eGFR at least 20 mL/min/1.73 m^2^
● Detection of dnDSA against the most recent kidney transplant with MFI >1000 at the latest annual HLA screening
Exclusion criteria:
● Patients younger than 18 years
● Patients unable or did not provide written informed consent
● Pregnant or breastfeeding persons
● Patients with increased bleeding risk
● Patients with multi-organ transplantation
● Patients who underwent kidney allograft biopsy after first detection of dnDSA
° Including those with biopsy-proven AMR
● Participation in another interventional clinical trial

dnDSA, de novo anti-HLA antibodies.

### Procedures

Study visits, including dd-cfDNA monitoring, were performed at study inclusion and after 1, 3, 6, 9 and 12 months in both groups. Study physicians informed patients and treating physicians about dd-cfDNA results for patients in the intervention group and withheld the information for patients in the control group until Month 12. Absolute dd-cfDNA values >50 copies/mL indicated a kidney allograft biopsy in the intervention group. Additionally, treating physicians could perform kidney allograft biopsy at any point during the study period in both groups for clinical indications such as rising creatinine or worsening proteinuria, but no definite thresholds were specified in the study protocol. Twelve months after study inclusion, kidney allograft biopsy was scheduled per protocol for patients who had not undergone kidney allograft biopsy until that point. At every study visit, a complete laboratory workup including complete blood count, kidney function parameters, dipstick urine and uACR, liver function parameters, C-reactive protein, BK viruria and viremia, and cytomegalovirus PCR was performed as part of clinical routine. Detailed methods of HLA-typing and dnDSA detection are described in Supplementary data, Item S1

### Donor-derived cell-free DNA, histopathology and Molecular Microscope Diagnostic System

Dd-cfDNA was collected at all study visits and immediately before biopsy, if the most recent measurement was >4 weeks ago. Each time, up to 16 mL of blood was drawn into two certified blood collection tubes (Streck Corp., Omaha, NE, USA) and shipped at ambient temperature to the processing laboratory within 3 days of collection. Dd-cfDNA was quantified using the droplet-digital PCR method as previously described and detailed in Supplementary data, Item S2 [32]. Test findings included both relative and absolute quantification of dd-cfDNA, with cutoffs of 0.5% and 50 copies/mL, as established in earlier validation trials [32, 33].

All biopsy specimens were read and interpreted during routine biopsy reporting according to the Banff 2019 classification by an experienced nephropathologist (K.A., M.B.-H.) who was unaware of the randomization status or the dd-cfDNA results [36]. Histological data were retrieved from the nephropathological reports. Along with conventional histopathological examination, additional analysis with the Molecular Microscope Diagnostic System (MMDx) was available for 23/26 biopsies [37, 38]. MMDx is a novel diagnostic tool using microarray technology to analyze the expression of various gene transcripts in allograft biopsies. For this purpose, an extra biopsy core was stored in RNALater (ThermoFisher Scientific, Henningsdorf, Germany) immediately after sample collection and shipped to the central laboratory Alberta Transplant Applied Genomics Center (ATAGC, University of Alberta, Edmonton, AB, Canada) at ambient temperature [37, 38]. RNALater is a non-toxic tissue storage reagent that rapidly permeates tissue and stabilizes and protects cellular RNA *in situ* in unfrozen specimens. For sensitivity analysis, all biopsies were reclassified according to the Banff 2022 classification by the study coordinator (B.O.) including the nephropathological reports and MMDx [39].

### Outcomes

The primary endpoint was the time from study inclusion to diagnosis of active AMR (aAMR) or chronic active AMR (caAMR). Secondary endpoints included time from first dnDSA occurrence to the diagnosis of AMR, diagnostic test metrics [sensitivity, specificity, positive predictive value, negative predictive value, area under the curve (AUC)] of longitudinal dd-cfDNA monitoring using a prespecified absolute cutoff of 50 copies/mL, an intra-individual increase of >25%, and a combination of both decision rules for the diagnosis of AMR. Additionally, clinical endpoints such as patient survival, death-censored graft survival, severe infection (infection leading to death, hospitalization or organ failure) and adverse events of kidney allograft biopsy, as well as laboratory values such as creatinine, eGFR (Chronic Kidney Disease Epidemiology Collaboration 2021), uACR and DSA MFI levels were recorded and analyzed (Supplementary data, Item S3) [40].

### Randomization

Patients were randomized 1:1 to dd-cfDNA-guided kidney allograft biopsy (intervention group) or clinician-guided biopsy (standard of care) over a period of 12 months. Randomization was stratified by time since the first occurrence of dnDSA (0–24 months vs ≥25 months). A simple randomization sequence was generated separately for both strata and concealed using sequentially numbered opaque envelopes by staff unrelated to the study investigation. Study physicians enrolled the patients, and the study coordinator (B.O.) performed allocation after patients provided consent. Patients, treating physicians and study personnel were aware of allocation (unblinded). Patients and treating physicians were blinded for dd-cfDNA results in the control group, while patients and treating physicians were aware of dd-cfDNA results in the intervention group.

### Statistical analysis

Sample size calculation is summarized in Supplementary data, Item S4 The demographics of the study participants are summarized as median and interquartile range (IQR) for continuous covariates or mean ± standard deviation (SD) for normally distributed parameters, and absolute and relative frequencies for categorical parameters. Statistically significant differences in the time from study inclusion or first dnDSA occurrence to AMR diagnosis between the two treatment arms were assessed using Wilcoxon test. Diagnostic test metrics were assessed using receiver operating characteristic analysis with prespecified decision thresholds (50 copies/mL and 25% increase to the 12 months mean dd-cfDNA) for all patients with kidney allograft biopsy as the gold standard. All secondary endpoint analyses were conducted in an exploratory fashion and no adjustment for multiple testing was performed. Correlation analysis was performed using Pearson correlation. Statistical tests were two-tailed and a *P*-value <.05 was considered statistically significant. Data analyses were performed using R, version 4.3.0 (R Foundation for Statistical Computing, Vienna, Austria).

## RESULTS

### Participants and clinical outcomes

We recruited 40 KTR with prevalent dnDSA, but without previous kidney allograft biopsy after first occurrence of dnDSA [median age 54.8 years; IQR 47.8–66.9; 33 (82.5%) men] (Table 2). Demographic and clinical baseline characteristics were generally similar across treatment groups (Table 2), except for HLA mismatch grade that was higher in the control group (median 4, IQR 3–4 vs median 3, IQR 2–3; *P* = .048).

**Table 2:** Baseline characteristics according to the treatment group.

	All participants	Intervention group	Control group
*N*	40	20	20
Median age in years (IQR)	54.8 (47.8–66.9)	57.5 (50.0–67.1)	52.4 (39.8–66.0)
Sex, *n* (%)			
Female	7 (17.5)	3 (15)	4 (20)
Male	33 (82.5)	17 (85)	16 (80)
Median BMI (IQR)	25.5 (22.3–29.2)	27.9 (24.0–29.6)	24.1 (21.6–26.4)
Median time on dialysis in years (IQR)	3.9 (1.0–7.4)	4.4 (1.2–6.7)	1.9 (1.0–8.1)
Median time since transplantation in years (IQR)	10.7 (4.2–15.1)	10.7 (4.3–13.7)	10.3 (4.2–15.7)
Preemptive transplantations, *n* (%)	10 (25)	5 (25)	5 (25)
Living donations, *n* (%)	22 (55)	12 (60)	10 (50)
Previous kidney transplantation, *n* (%)	1 (2.5)	0 (0)	1 (5)
Induction immunosuppression, *n* (%)			
Basiliximab	34 (85)	18 (90)	16 (80)
Maintenance immunosuppression, *n* (%)			
Steroid	23 (57.5)	13 (65)	10 (50)
MPA	38 (95)	18 (90)	20 (100)
Tacrolimus	23 (57.5)	11 (55)	12 (60)
Cyclosporin	12 (30)	6 (30)	6 (30)
HLA-mismatch grade, median (IQR)	3 (2–4)	3 (2–3)	4 (3–4)
PIRCHE-II sum score, median (IQR)	68.9 (55.8–98.9)	62.4 (46.0–97.7)	73.7 (60.3–111.8)
cPRA, *n* (%)			
0%	37 (92.5)	19 (95)	18 (90)
0%–25%	3 (7.5)	1 (5)	2 (10)
			
Previous borderline rejection or TCMR episode, *n* (%)	8 (20)	3 (15)	5 (25)
			
Median months since first dnDSA occurrence (IQR)	24.9 (7.2–114.3)	24.0 (7.2–96.5)	33.5 (12.2–116.7)
Multiple dnDSA, *n* (%)	14 (35)	6 (30)	8 (40)
Class 1 dnDSA only, *n* (%)	5 (12.5)	2 (5)	3 (7.5)
Class 2 dnDSA only, *n* (%)	27 (67.5)	15 (37.5)	12 (30)
Class 1 + 2 dnDSA, *n* (%)	8 (20)	3 (7.5)	5 (12.5)
MFI at baseline, median (IQR)	4685 (1627–16 974)	3855 (1472–9565)	6809 (2420–19 550)
Median eGFR in mL/min/1.73 m^2^ at Day 0 (IQR)	51.4 (40.3–62.0)	47.2 (37.4–59.5)	54.4 (46.4–62.8)
eGFR slope in the previous year in mL/min/1.73 m^2^/year	–0.16 ± 2.29	–0.61 ± 2.43	0.28 ± 2.11
eGFR slope in the previous 2 years in mL/min/1.73 m^2^/year	–0.71 ± 2.36	–1.23 ± 2.36	−0.18 ± 2.29
Median uACR in mg/g at Day 0 (IQR)	55 (22–276)	62 (30–263)	52 (22–286)

MPA, mycophenolic acid; cPRA, calculated panel reactive antibody.

By the end of the study, 39/40 patients had functioning grafts, and one patient in the control group died unrelated to the study. No cases of BK viremia occurred. Four patients (two in each group) experienced adverse events due to transplant biopsy (one hematuria, one bladder tamponade, and two arteriovenous fistulas), none of which led to persistent morbidity or impairment of kidney function. Clinical outcomes during the study did not differ between both groups as shown in Supplementary data, Item S5. Of the 39 patients with a follow-up measurement of dnDSA MFI, 7 patients (18%) had an MFI reduction to <50% of the baseline MFI, and two patients (5%) had an increase in MFI >100%, while the majority of 30 patients (77%) had persistent dnDSA MFI. After study completion, all patients were followed for 24 months after baseline. Treatment and 24 months follow-up data for patients with biopsy-proven AMR were summarized as Supplementary data, Table S1.

From 39 patients that completed the last visit, 16 had increased absolute dd-cfDNA from which 13 (81%) underwent kidney biopsy (intervention group: 7/8—all for increased dd-cfDNA; control group: 6/8—all for protocol biopsy). From the remaining 23 patients without increased dd-cfDNA, 13 (57%) underwent kidney biopsy (intervention group: 6/12—3 for clinical indication, 3 for protocol biopsy; control group: 7/11—2 for clinical indication, 5 for protocol biopsy). In total, 26/39 (67%) patients with complete follow-up underwent kidney biopsy, and 13/39 (33%) withdrew consent for protocol biopsy. Three patients, who refused protocol biopsy had increased dd-cfDNA that was indicative of underlying AMR (Supplementary data, Fig. S1).

In total, 12/26 (46.2%) patients that underwent biopsy had biopsy-proven AMR. From these, four (33%) had stable kidney function (uACR ≤300 mg/g and max. creatinine increase ≤0.3 mg/dL) and were classified as subclinical AMR. All patients with subclinical AMR had increased levels of dd-cfDNA (range 57–161 copies/mL), and increased molecular AMR scores (range 0.48–0.8; reference range <0.2).

### Primary endpoint: time from study inclusion to diagnosis of antibody-mediated rejection

The primary endpoint was assessed only in patients with biopsy-proven AMR. In the intervention group, seven patients had biopsy-proven AMR, which was detected after a median of 2.8 months (IQR 1.7–5.3) after study inclusion. In the control group, five patients had biopsy-proven AMR, which was detected after a median of 14.5 months (IQR 13.3–16.7) after study inclusion. The time from study inclusion to biopsy-proven AMR was significantly shorter in the intervention group than in the control group (*P* = .003).

**Figure 2:**
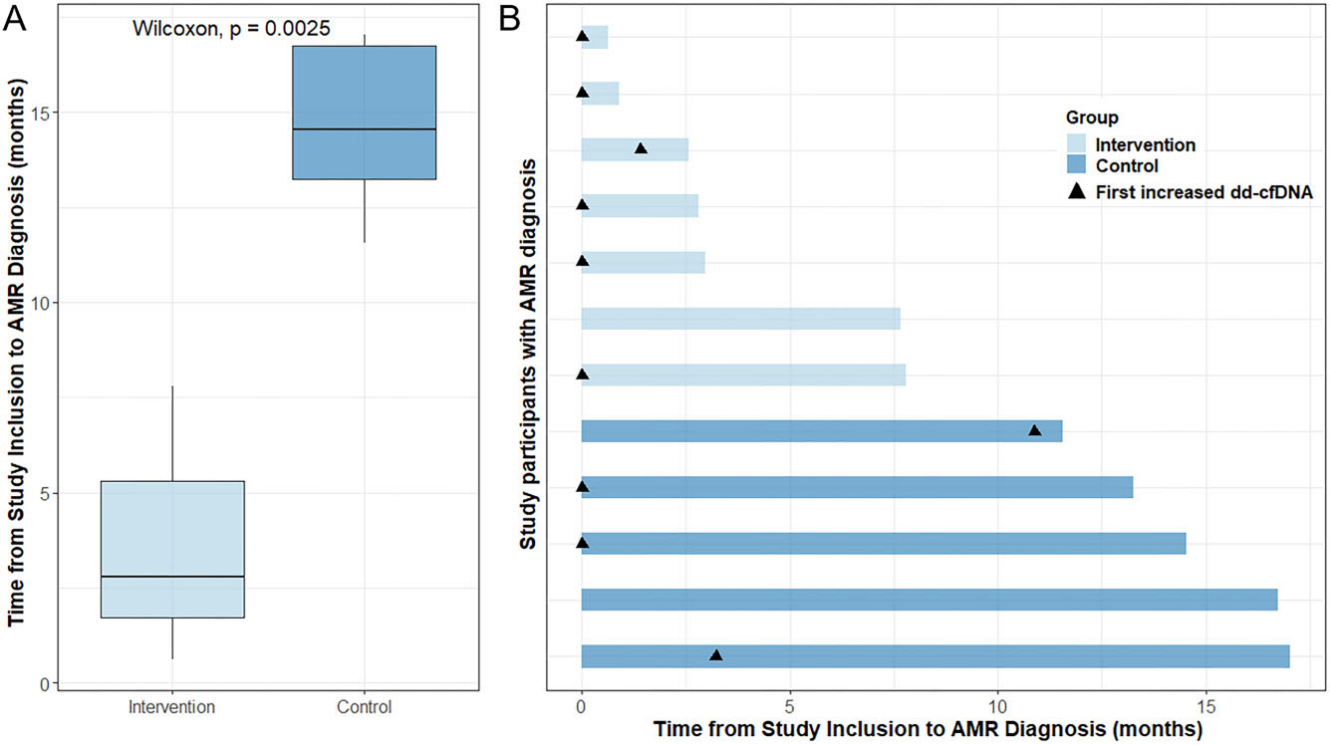
(**A**) Boxplots comparing the time from study inclusion to AMR diagnosis for patients with AMR according to treatment groups. (**B**) Time from study inclusion to AMR diagnosis in months for each patient with AMR diagnosis according to treatment group. Arrowheads indicate the timing of the first increase in absolute dd-cfDNA above the prespecified cutoff of 50 copies/mL for each patient, and no arrowheads indicate absolute dd-cfDNA below the prespecified cutoff over the observation period.

Figure 2 summarizes the time to AMR diagnosis for each patient and indicates the timing of the first increase in dd-cfDNA measurement for each patient. The delays observed from first increase in dd-cfDNA to biopsy in the intervention group as well as the delays from 12 months to protocol biopsy in the control group were caused by need for scheduling the biopsy, or concurrent medical diagnoses. A sensitivity analysis indicates that ideal timing of biopsies (using the date of first increased dd-cfDNA or date of clinical indication biopsy in the intervention group, and the date of clinical indication biopsy or 12 months after inclusion in the control group) would still have resulted in a significant difference in time to diagnosis (*P* = .0035, Supplementary data, Item S6). As indicated in Fig. 2, dd-cfDNA-guided kidney biopsy could have reduced the time to AMR diagnosis by >6 months in 3/5 patients (60%).

### Secondary endpoints

#### Time from *de novo* DSA occurrence to diagnosis of antibody-mediated rejection

No difference was found between time since first dnDSA occurrence and AMR diagnosis between intervention and control group (median 22.1 months, IQR 12.1–90.1 vs median 120.2 months, IQR 54.1–142.7; *P* = .25).

#### Biopsy results and donor-derived cell-free DNA results

The biopsy results obtained per group and biopsy indication are provided in Table 3, together with the dd-cfDNA results at the time of biopsy for each diagnosis. Dd-cfDNA was higher in AMR in comparison with CNI toxicity or unremarkable histopathology both in absolute (*P* = .004 and *P* = .007, respectively) and relative (*P* = .045 and *P* = .002, respectively) quantification.

**Table 3:** Biopsy results and indication for each treatment group and the respective dd-cfDNA results at the time of biopsy.

Group	Biopsy indication	*n*	aAMR	caAMR	TCMR	Unremarkable	CNI toxicity	Other
Intervention	dd-cfDNA >50 copies/mL	7	5	1			1	
	Clinical	3	0	1			1	1
	Protocol	3	0	0		1	1	1
Control	Clinical	2	0	0	1			1
	Protocol	11	2	3		4	2	
Total count			7	5	1	5	5	3
Median absolute dd-cfDNA, copies/mL (IQR)			63 (59–92)	39 (18–67)	30	10 (9–16)	14 (12–16)	8 (range 6–34)
Median relative dd-cfDNA, % (IQR)			1.31 (0.97–2.52)	2.14 (1.10–3.80)	0.4	0.44 (0.29–0.58)	0.78 (0.5–0.8)	0.33 (range 0.28–0.7)

AMR, antibody-mediated rejection; TCMR, T-cell mediated rejection; CNI, calcineurin inhibitor; IQR, interquartile range.

Individual longitudinal absolute dd-cfDNA measurements are shown in Supplementary data, Fig. S2A for patients with AMR and Supplementary data, Fig. S2B for patients with non-AMR histopathological diagnoses.

At baseline, 7/12 patients (58.3%) with AMR had dd-cfDNA above 50 copies/mL, and 0/14 patients without AMR. Over the study period, six additional patients had increases in absolute dd-cfDNA >50 copies/mL, three of whom had AMR in the biopsy. Regarding relative quantification of dd-cfDNA, 21/26 patients had dd-cfDNA above the cutoff of 0.5% over the study period, only 12 of whom had AMR in the biopsy (Supplementary data, Fig. S3).

### Diagnostic test metrics of donor-derived cell-free DNA vs routine biomarkers

Longitudinal dd-cfDNA monitoring with an absolute cutoff of 50 copies/mL had following test metrics for the detection of AMR in DSA+ patients: AUC, 0.92; sensitivity, 0.83; specificity, 0.79; positive predictive value (PPV), 0.77; negative predictive value (NPV), 0.85 (Table 4). Using intra-individual increases in absolute dd-cfDNA or combining an absolute cutoff with an intra-individual increase did not improve diagnostic test metrics (Supplementary data, Item S7 and Tables S2–S4).

**Table 4:** Contingency table and diagnostic test metrics of longitudinal absolute dd-cfDNA monitoring with a cutoff of 50 copies/mL for AMR in DSA + KTR.

	AMR	No AMR	Total	
>50 copies/mL	10	3	13	PPV 0.77
≤50 copies/mL	2	11	13	NPV 0.85
Total	12	14	26	Prev 0.46
	Sens 0.83	Spec 0.79		Acc 0.81

Prev, prevalence of AMR; Acc, accuracy; Sens, sensitivity; Spec, specificity.

Relative quantification did not differ from absolute quantification in terms of AUC (0.91 vs 0.92, *P* = .86), but resulted in lower specificity and higher sensitivity when using the prespecified cutoff of 0.5% (sensitivity, 1.0; specificity, 0.36; PPV, 0.57; NPV, 1.00; Supplementary data, Table S5). Routine biomarkers such as uACR, creatinine and DSA MFI showed poor diagnostic test metrics for detection of AMR (Tables 5–7, Supplementary data, Item S8 and Tables S6–S8)

**Table 5:** Contingency table and diagnostic test metrics of longitudinal uACR monitoring with a cutoff of 300 mg/g for AMR in DSA + KTR.

	AMR	No AMR	Total	
>300 mg/g	7	4	11	PPV 0.54
≤300 mg/g	5	10	15	NPV 0.77
Total	12	14	26	Prev 0.46
	Sens 0.58	Spec 0.71		Acc 0.65

Prev, prevalence of AMR; Acc, accuracy; Sens, sensitivity; Spec, specificity.

**Table 6:** Contingency table and diagnostic test metrics of >0.3 mg/dL increase in creatinine from baseline creatinine for AMR in DSA + KTR.

	AMR	no AMR	Total	
>0.3 mg/dL	3	4	7	PPV 0.43
≤0.3 mg/dL	9	10	19	NPV 0.53
Total	12	14	26	Prev 0.46
	Sens 0.25	Spec 0.71		Acc 0.5

Prev, Prevalence of AMR; Acc, accuracy; Sens, sensitivity; Spec, specificity.

**Table 7:** Contingency table and diagnostic test metrics of MFI doubling from first occurrence to maximum MFI for AMR in DSA + KTR.

	AMR	No AMR	Total	
MFI increase >100%	5	2	7	PPV 0.71
Stable or decreasing MFI	7	12	19	NPV 0.63
Total	12	14	26	Prev 0.46
	Sens 0.42	Spec 0.86		Acc 0.65

Prev, Prevalence of AMR; Acc, accuracy; Sens, sensitivity; Spec, specificity.

### Correlation of histopathology, Molecular Microscope Diagnostic System and donor-derived cell-free DNA

**Figure 3:**
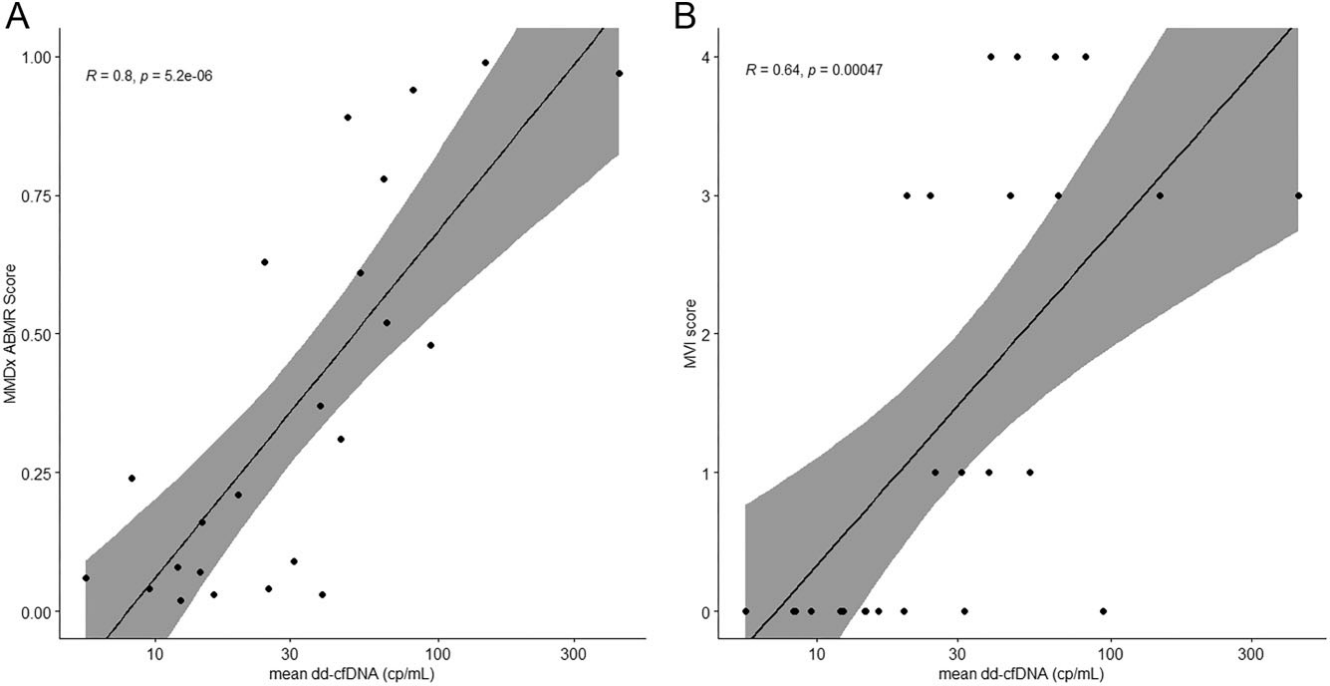
(**A**) Scatterplot showing the Pearson correlation coefficient between log-transformed mean dd-cfDNA in copies/mL and the molecular AMR score. (**B**) Scatterplot showing the Pearson correlation coefficient between log-transformed mean dd-cfDNA in copies/mL and the microvascular inflammation score (g + ptc) in conventional histopathology.

After log-transformation to adjust for skewing, mean absolute dd-cfDNA showed strong correlation (R = 0.8, *P* < .001, Fig. 3A) with the molecular AMR score (antibody-mediated rejection probability classifier, ABMRpm), the total rejection score (R = 0.79, *P* < .001, Supplementary data, Fig. S4A) and the inflammation score (R = 0.74, *P* < .001, Supplementary data, Fig. S4B). It showed moderate correlation with the acute kidney injury score (R = 0.52, *P* = .01, Supplementary data, Fig. S4C), which is based on injury-repair response–associated transcripts (IRRATs), but no significant correlation with the fibrosis score (R = 0.31, *P* = .15, Supplementary data, Fig. S4D) or the T cell-mediated rejection (TCMR) score (R = 0.26, *P* = .23, Supplementary data, Fig. S4E), the latter of which was due to absence of molecular TCMR with all scores <0.1. Furthermore, mean absolute dd-cfDNA showed a moderate correlation with histological AMR features such as microvascular inflammation (g + ptc) (R = 0.64, *P* < .001, Fig. 3B). Correspondingly, we found higher molecular AMR scores in patients undergoing biopsy due to increased dd-cfDNA levels than in those undergoing protocol biopsies.

AMR biopsies detected in the intervention group showed higher molecular AMR probability than those in the control group (ABMRpm: median 0.92; IQR 0.81–0.96 vs median 0.52; IQR 0.31–0.61; *P* = .03), while there was no significant difference in histological activity assessed by activity index (g + ptc + C4d + v) or MVI score (g + ptc) between both groups (median 4, IQR 3–5 vs median 4, IQR 3–4; *P* = .45; median 3, IQR 3–4 vs median 3, IQR 3–3; *P* = .60). There was no difference in Early AMR transcripts between intervention and control group in AMR biopsies (median 0; range 0–0.33 vs median 0; range 0–0.82; *P* = .91) (Supplementary data, Item S9).

### Banff classification 2022

After reclassification of kidney biopsies according to Banff 2022 classification including MMDx, 2 patients could be classified as (probable) AMR, who have previously been classified as non-AMR (case details in Supplementary data, Item S10). After accounting for these changes, there was still a significant difference in time to AMR diagnosis between intervention and control group (median 2.9 months; IQR 2.1–6.2 vs median, 14.4 months; IQR 13.5–16.2; *P* < .001). The test characteristics of longitudinal dd-cfDNA monitoring improved as follows: AUC, 0.96; sensitivity, 0.86; specificity, 0.92; PPV, 0.92; NPV, 0.85 (Supplementary data, Table S9).

## DISCUSSION

We had hypothesized that longitudinal dd-cfDNA monitoring and dd-cfDNA-guided biopsy can reduce the time to AMR diagnosis in patients with prevalent dnDSA, but without previous biopsy after dnDSA occurrence. Our study shows that AMR diagnosis is made significantly earlier in patients that were randomized to dd-cfDNA-guided biopsy compared with those randomized to clinician-guided biopsy, which supports this hypothesis. Furthermore, we observed that absolute dd-cfDNA was increased in several patients with subclinical AMR. Our findings underscore the latest results from the ProActive study, a prospective, observational registry study, which has shown that dd-cfDNA was significantly elevated 5 months before histological diagnosis of AMR, with 85% of biopsies being for cause [41].

To our knowledge, this is the first randomized clinical trial to investigate the potential benefit of dd-cfDNA monitoring after kidney transplantation. While early diagnosis is not beneficial as long as efficacious therapies for AMR are lacking, this might change with emerging candidate drugs such as CD38 targeting agents, which showed promising results for the treatment of AMR [17]. Consequently, dd-cfDNA-guided early diagnosis may become increasingly important, since timely therapeutic intervention could significantly change the course of AMR by preventing chronic irreversible damage and thereby improve outcomes.

We also confirmed the high sensitivity and specificity of dd-cfDNA for AMR as observed in previous studies. However, we found a higher PPV of 77% compared with the 12%–61% reported in earlier cohorts [27, 33, 34, 42]. This is as anticipated regarding the selection of dnDSA positive patients and, consequently, higher pre-test probability for AMR. Importantly for clinical practice, dd-cfDNA detected significantly more patients with AMR than routine biomarkers, such as serum creatinine or urine albumin, which had low AUC similar to previous studies [31]. Likewise, indicators of alloimmune response, such as PIRCHE-II and dnDSA-MFI (including MFI sum and maximum MFI of the immunodominant DSA), were inferior to dd-cfDNA in distinguishing AMR.

Another strength of our study is that it demonstrated the diagnostic accuracy of both absolute and relative dd-cfDNA, providing notable evidence for the relevance of the quantification method. AUC did not differ between absolute and relative dd-cfDNA levels; however, we observed important differences in the dynamics of each course that need to be discussed. Serial assessment of absolute dd-cfDNA showed increased sensitivity for AMR compared with a single measurement at study inclusion, since several patients with AMR, despite normal baseline dd-cfDNA levels, showed strong increases over time, supporting the need for longitudinal monitoring. Although specificity was reduced with longitudinal assessment, increases in three non-AMR patients still indicated therapy-requiring biopsy results. For relative quantification, the results should be interpreted cautiously because we observed false-positive elevations during the study period in a high proportion of KTR without evidence of AMR or other injuries in the subsequent biopsy. This is reflected by the low PPV (0.57) for relative quantification and justifies previously raised concerns that dd-cfDNA monitoring might increase the number of non-informative biopsies [43]. In view of these critical observations, our interpretation is that the prespecified cut-off of 0.5% for relative dd-cfDNA may not be ideal for patients in the late post-transplant period. This uncertainty regarding the cutoff for relative quantification has been raised previously by Schütz *et al*. and could be explained by a decline in the levels of recipient cfDNA over time, which leads to an apparent increase in relative dd-cfDNA [44]. Additionally, relative quantification is more error prone due to fluctuations in the recipients cfDNA than absolute quantification of dd-cfDNA, which should also be noted [45].

It is important to note that test metrics of dd-cfDNA improved when biopsies were reclassified according to the latest Banff 2022 classification. This can be explained by additional AMR diagnoses based on molecular diagnostics and the strong correlation between absolute dd-cfDNA levels and molecular AMR transcripts in MMDx, which we demonstrated in agreement with previous studies [46–48]. From a clinical perspective, dd-cfDNA elevation can help to confirm suspected AMR in cases of ambiguous histology by triggering additional MMDx analysis, while decrease in dd-cfDNA can indicate therapy response, as demonstrated in the felzartamab trial [17].

### Limitations

This was a single-center, open-label study with a small sample size, and every third participant did not undergo kidney allograft biopsy by the end of the observation period. In some cases, biopsy was delayed due to the need to schedule the biopsy, or concurrent medical diagnoses. Also, there were no prespecified decision thresholds for routine biomarkers that led to clinical indication biopsy. The randomization resulted in higher HLA-mismatch grade in the control group, which could potentially lead to worse outcomes in the control group. However, all patients included in the study already had dnDSA at the time of study inclusion, which limits the importance of HLA-mismatch.

Only one biopsy showed TCMR, which showed discrepancy between MMDx and histology, and no biopsy showed co-occurrence of TCMR and AMR, which is an otherwise common entity in cross-sectional studies of kidney allograft biopsies. Thus, we were unable to evaluate the performance of dd-cfDNA either in the case of mixed rejection or in distinguishing between the two rejection types. Additionally, advanced transplant age in this cohort reduced the likelihood of early post-transplant graft dysfunction, such as ischemia–reperfusion injury, delayed graft function and infection. This could potentially increase the diagnostic performance due to the absence of alternative reasons for increased dd-cfDNA [49, 50]. Other injury patterns that could potentially interfere with dd-cfDNA, such as BK virus–associated nephropathy (BKVAN), were not present in our cohort [51].

## CONCLUSION

Dd-cfDNA monitoring using absolute quantification and dd-cfDNA-guided biopsy in KTR with prevalent dnDSA can reduce the time to diagnosis of active or chronic active AMR in comparison with clinician-guided biopsy and can identify subclinical forms of AMR. Confirmation in larger multicenter studies including patients with incident dnDSA is needed.

## Supplementary Material

gfae282_Supplemental_File

## Data Availability

Deidentified, individual participant data underlying the results presented in the manuscript will be available for researchers, who provide a reasonable request to the corresponding author at aylin.akifova@charite.de immediately following publication.
