# Supplementary material for: Donor-derived cell-free DNA monitoring for early diagnosis of antibody-mediated rejection after kidney transplantation: a randomized trial
Source: Nephrol Dial Transplant. 2024 Nov 29;40(7):1384–95. doi: 10.1093/ndt/gfae282 (PMC12207606; doi:10.1093/ndt/gfae282)
Supplement: gfae282_Supplemental_File [file gfae282_supplemental_file.docx]

**Supplementary Material**

**Supplemental Item 1**: Detailed description of HLA-typing methods and dnDSA detection.

**Supplemental Item 2.** Detailed method description for donor-derived cell-free DNA quantification.

**Supplemental Item 3.** Detailed secondary endpoint definitions.

**Supplemental Item 4.** Sample size calculation.

**Supplemental Item 5.** Clinical outcome parameters including eGFR slope, uACR, hospitalization and infection.

**Supplementary Item 6**. Sensitivity analysis comparing “ideal” time from study inclusion to diagnosis between both groups.

**Supplemental Item 7.** Detailed analysis of dd-cfDNA test metrics with prespecified decision thresholds.

**Supplemental Item 8.** Analysis of routine biomarkers, dnDSA mean fluorescence intensity (MFI) and PIRCHE-II sum scores for the detection of AMR.

**Supplemental Item 9.** In-depth analysis of histopathology and molecular rejection phenotypes in patients with biopsy showing antibody-mediated rejection (AMR).

**Supplemental Item 10.** In-depth analysis of cases that were reclassified as AMR or probable AMR according to Banff 2022 classification.

**Supplemental Figure 1.** Absolute donor-derived cell-free DNA in 14 patients without kidney biopsy.

**Supplemental Figure 2.** Absolute donor-derived cell-free DNA in **A)** patients with biopsy-proven AMR, grouped by the time since first DSA occurrence according to the strata used for randomization (0-24 months vs. ≥ 25 months). **B)** patients with non-AMR histopathological diagnosis, grouped by the time since first DSA occurrence according to the strata used for randomization (0-24 months vs. ≥ 25 months)

**Supplemental Figure 3.** Relative donor-derived cell-free DNA in **A)** patients with biopsy-proven AMR, grouped by the time since first DSA occurrence according to the strata used for randomization (0-24 months vs. ≥ 25 months). **B)** patients with non-AMR histopathological diagnosis, grouped by the time since first DSA occurrence according to the strata used for randomization (0-24 months vs. ≥ 25 months)

**Supplemental Figure 4.** Pearson correlation of log transformed mean absolute dd-cfDNA with different molecular scores in MMDx.

**Supplemental Table 1.** Follow-up data from patients with biopsy-proven antibody-mediated rejection (AMR) during the study period including treatments and key clinical and laboratory outcomes.

**Supplemental Table 2.** Contingency table and diagnostic test metrics of maximum intraindividual increase of > 25% compared to the 12 months mean in absolute dd-cfDNA for AMR in DSA+ KTR.

**Supplemental Table 3.** Contingency table and diagnostic test metrics of a combined criterium of longitudinal absolute dd-cfDNA monitoring with a cutoff of 50 copies/mL or maximum intraindividual increase of > 25% compared to the 12 months mean in absolute dd-cfDNA for AMR in DSA+ KTR.

**Supplemental Table 4.** Contingency table and diagnostic test metrics of baseline absolute dd-cfDNA with a cutoff of 50 copies/mL for AMR in DSA+ KTR.

**Supplemental Table 5.** Contingency table and diagnostic test metrics of longitudinal relative dd-cfDNA monitoring with a cutoff of 0.5 % for AMR in DSA+ KTR.

**Supplemental Table 6.** Contingency table and diagnostic test metrics of longitudinal uACR monitoring with a cutoff of 1000 mg/g for AMR in DSA+ KTR

**Supplemental Table 7.** Contingency table and diagnostic test metrics of MFI doubling from baseline to study completion for AMR in DSA+ KTR.

**Supplemental Table 8.** Contingency table and diagnostic test metrics of MFI halving from baseline to study completion for AMR in DSA+ KTR.

**Supplemental Table 9.** Contingency table and diagnostic test metrics of longitudinal absolute dd-cfDNA monitoring with a cutoff of 50 copies/mL for AMR in DSA+ KTR according to Banff 2022 classification with probable AMR classified as AMR.

**Supplemental Item 1**: Detailed description of HLA-typing methods and dnDSA detection.

Recipients were routinely typed for HLA by sequence-specific oligonucleotides (SSO) before 2018 and next generation sequencing (NGS) thereafter. Postmortem kidney donors were routinely HLA typed by sequence-specific primers (SSP) at the donor center and retyping was performed by sequence-specific oligonucleotides (SSO) before 2018 and next generation sequencing (NGS) thereafter at our center. Living kidney donors were routinely typed for HLA by sequence-specific oligonucleotides (SSO) before 2018 and next generation sequencing (NGS) thereafter. The dnDSA were mostly defined on the antigen level, however, when necessary, HLA allele information has been considered in assigning DSA appropriately. Therefore, retyping by NGS was performed in some instances. If necessary for the assignment of DSA, 4-digit HLA typing by NGS was performed after 2018. Before 2018, SSO was performed and the most probable 4-digit result according to CWD 2.0 filter was considered.

Screening for donor specific antibodies anti-HLA antibodies (DSA) was performed using LuminexⓇ -based LABScreenⓇ SAB assay (One Lambda, Canoga Park, CA), as widely standardized and described previously.^1^ Standard-of-care crossmatch testing for anti-HLA-antibodies was performed in all subjects pretransplant and de novo DSA were detected by routine annual screening. Antibodies with a mean florescence intensity (MFI) above the threshold of 1000 in LuminexⓇ were considered positive and the immunodominant DSA was defined as the DSA with the highest MFI value in the most recent anti-HLA-DSA test prior to biopsy. Further, MFI values at first occurrence of dnDSA, as well as the peak MFI over time were assessed until the time of index biopsy. Complementary testing for additional DSA-characteristics, such as IgG-subclass or complement-fixing ability, as well as screening non-HLA antibodies was not performed for the purpose of this study. The results of every HLA-sample testing were reviewed, confirmed and signed off by local histocompatibility experts in our hospital (N.L., D.St.) before delivering the results for clinical evaluation.

**Supplemental Item 2.** Detailed method description for donor-derived cell-free DNA quantification.

For each patient, four informative single nucleotide polymorphisms (SNPs), defined as SNPs for which the recipient is homozygous and the donor carries at least one heterozygous allele, were selected from a set of 40 predefined SNPs. These four SNPs were used to quantify the dd-cfDNA concentration (%) as follows: donor alleles/(donor alleles + recipient alleles). The results for SNPs with heterozygous donor genotypes were corrected by a factor of 2. Total cfDNA was extracted from up to 8 mL of plasma, and the concentration was determined as haploid genome copies using droplet-digital PCR and the result was corrected for extraction loss and cfDNA fragmentation, as described previously.^1^ The absolute concentration of dd-cfDNA (copies/mL plasma) was calculated by multiplying total cfDNA (copies/mL) and dd-cfDNA (%). Reference ranges for total cfDNA in the post-transplant course were previously established in a cohort of 300 KTR.^2^

**Supplemental Item 3.** Detailed secondary endpoint definitions.

The eGFR was calculated from creatinine according to the CKD-EPI 2021 formula.^3^ A 1-year eGFR slope was calculated with all available eGFR values from day 0 to month 12 using a linear mixed-effects model with random intercepts and slopes to estimate the slope for each individual. Using the same approach, yearly baseline eGFR slope was calculated for the year before study inclusion and for the 2 years before study inclusion, based on all available outpatient creatinine values for the respective timeframes. Baseline creatinine was defined as the mean of all outpatient creatinine values in the year before study inclusion. Clinical stability was defined as a maximum increase in creatinine ≤0.3 mg/dL above the baseline creatinine and maximum uACR ≤300 mg/g at any time during the observation period.

To reflect the dynamics of dnDSA, we studied antibody properties including alterations in HLA-class, number, specificity and antibody strength assessed by MFI evolution over time. For the latter, we selected the immunodominant DSA at inclusion and analyzed its MFI values at first occurrence, maximum MFI at any time, MFI at study inclusion and MFI at the end of the study period. Patients with MFI-fluctuations in the range of -50%/+100% were defined as persistent. Increases of more than 100% MFI were considered as clinically relevant, whereas decline in dnDSA intensity was defined by MFI reduction of >50% or temporary negativity (MFI below positivity cutoff of 1000). Further, we evaluated the MFI sum values at study inclusion, as well as at the time of biopsy. PIRCHE-II scores were calculated as previously described.^4^

**Supplemental Item 4.** Sample size calculation.

The sample size was calculated based on the following assumptions. A Wilcoxon test was planned to compare the time from study inclusion to AMR diagnosis in patients with biopsy-proven AMR at the end of the study period between intervention and control group. In patients with biopsy-proven AMR, we estimated that kidney biopsy will be performed 3 months (± 3 months) after study inclusion in the intervention group and 9 months (± 3 months) after study inclusion in the control group. Based on these assumptions, 6 patients with biopsy-proven AMR in each group would be sufficient to reach a power of 80% with an alpha error of 0.05. Based on data from Schinstock et al. ^5^, we estimated that approximately 50% of patients will have an underlying AMR during the study period. Since the sensitivity of dd-cfDNA for detection of AMR is between 0.73 and 1.0 throughout various studies ^1,6,7^, and patients in both groups will be informed about dd-cfDNA results at least after 12 months, it can be assumed that 80% of patients with underlying AMR will actually be diagnosed with AMR at the end of the study (40% of the study population). This results in a minimum sample size of 30 patients. To account additional for an additional proportion of patients that withdraw consent before protocol biopsy despite increased dd-cfDNA values, we determined a total sample size of 40 patients.

**Supplemental Item 5.** Clinical outcome parameters including eGFR slope, uACR, hospitalization and infection.

The eGFR slope during the observation period was -0.29±1.14 ml/min/1.73 m^2^ per year within our cohort and did not differ significantly between intervention (-0.03±0.94 ml/min/1.73 m^2^ per year) and control (-0.56±1.29 ml/min/1.73 m^2^ per year, p=0.15). uACR did not differ between the intervention (median 67 mg/g, IQR 24-249) and control (median 74 mg/g, IQR 30-264, p=0.96) groups at the end of the observation period.

Five patients were hospitalized due to infection during the observation period: two patients in the control group and three patients in the intervention group (p=1.00).

**Supplementary Item 6**. Sensitivity analysis comparing “ideal” time from study inclusion to diagnosis between both groups.

We also performed a sensitivity analysis to test, if the effect seen would be present if the timing of biopsies would have been ideal. Therefore, for the intervention group, we used the first of the following: date of the first increased dd-cfDNA sample, date of clinical indication biopsy, 12 months after inclusion, and for the control group the first of the following: date of clinical indication biopsy or 12 months after inclusion. Using these dates, we calculate an “ideal” time to biopsy in patients with AMR diagnosis and compared these times between intervention and control with a Wilcoxon test. There was still a significant difference in time to AMR diagnosis in this sensitivity analysis (Intervention: median 0 months [IQR 0-0.71], Control: median 12 [IQR 12-12], p=0.003522). Consequently, perfect timing of biopsies would not have changed the main results of the study.

**Supplemental Item 7.** Detailed analysis of dd-cfDNA test metrics with prespecified decision thresholds.

Longitudinal dd-cfDNA monitoring with a maximum intra-individual increase of >25% compared with the mean in absolute dd-cfDNA during the observation period, irrespective of absolute values, was a poor predictor for the detection of AMR in DSA+ patients (AUC, 0.3; sensitivity, 0.5; specificity, 0.14; PPV, 0.33; NPV, 0.25; Supplemental Table 1).

Consequently, combining both decision rules resulted in a slight increase in sensitivity to 1.0 at the expense of strongly reduced specificity of 0.14, resulting in a PPV of 0.5, and NPV of 1.0 (Supplemental Table 2).

Using only the first dd-cfDNA value, the AUC was 0.89, which was not different from longitudinal monitoring (AUC 0.92, p=0.61). When compared to longitudinal monitoring, each with an absolute cutoff of 50 copies/mL, this approach resulted in lower sensitivity and NPV of 0.58 and 0.74, respectively, but higher specificity and PPV of 1.0 each (Supplemental Table 3).

**Supplemental Item 8.** Analysis of routine biomarkers, dnDSA mean fluorescence intensity (MFI) and PIRCHE-II sum scores for the detection of AMR.

Maximum uACR (AUC 0.59) and maximum increase from baseline creatinine (AUC 0.6) during the observation period showed lower AUC for AMR detection than longitudinal dd-cfDNA monitoring (AUC 0.92, p=0.02, and p=0.003, respectively). Diagnostic test metrics for uACR with cutoffs of 300 mg/g and 1000 mg/g are provided in Table 5 and Supplemental Table 5, respectively, and diagnostic test metrics for creatinine increase >0.3 mg/dL in Table 6.

For MFI at baseline, the AUC was 0.58, and a cutoff determined by the Youden point (MFI=1543) resulted in the following test metrics: sensitivity, 0.92; specificity, 0.43; PPV, 0.58; and NPV, 0.86. For maximum MFI, AUC was 0.55, and a cutoff determined by the Youden point (MFI=10160) resulted in the following test metrics: sensitivity, 0.58; specificity, 0.64; PPV, 0.58; NPV, 0.64. Both MFI at baseline and maximum MFI had a lower AUC than dd-cfDNA at baseline for the detection of AMR (p<0.009 and p=0.014, respectively). MFI doubling from first occurrence to maximum MFI showed the following test metrics: sensitivity, 0.42; specificity, 0.86; PPV, 0.71, NPV 0.63 (Table 7). Additionally, dnDSA MFI sum did not differ between patients with biopsy-proven AMR and patients without AMR in biopsy (at biopsy: median 6377, IQR 1805 - 20433 vs. median 5740, IQR 1550 - 19892, p=0.63; at baseline: median 7004, IQR 3741 - 22352 vs. median 8562, IQR 2178 - 20291, p=0.78). Also, the frequency of AMR did not differ considerably based on MFI sum category (low 1000-2999: 40% AMR, intermediate: 3000-5999: 40%, high >=6000: 50%), which is line with the results of the comparison tests.^7^

The PIRCHE-II sum scores were significantly higher in patients with AMR (median 74.1, IQR 47.7 - 106.8) than in patients with unremarkable biopsy (median 63.4, IQR 57–68.9, p = 0.003) or CNI toxicity (median 52.9, IQR 50–56.8, p=0.002). The AUC of the PIRCHE-II sum score for AMR detection was 0.57.

**Supplemental Item 9.** In-depth analysis of histopathology and molecular rejection phenotypes in patients with biopsy showing antibody-mediated rejection (AMR).

From 7 cases of AMR in the intervention group, 5 showed active AMR (aAMR) and 2 showed chronic-active AMR (caAMR) - 1 with cg2 and 1 with cg1a. From 5 cases of AMR in the control group, 2 showed aAMR and 3 showed caAMR (with cg1, cg2, and cg3). However, there was no statistically significant difference in the frequency of patients with caAMR, cg score or chronicity index (ci+ct+cv+cg*2) between both groups.

Regarding MMDx rejection phenotypes, AMR biopsies in the intervention group predominantly showed R5 (Fully-developed-ABMR) phenotype (with a median R5 score of 0.75, IQR 0.68-0.91), only few transcripts from the R4 (Early-ABMR) phenotype (median score 0, IQR 0-0.18) with 2 patients with 0.25 and 0.33 R4 score, and almost no transcripts from the R6 (Late-stage-ABMR) phenotype (median 0.02, IQR 0-0.08). The AMR-biopsies in the control group showed more heterogenous MMDx rejection phenotypes. One patient showed an R4 phenotype (Early-ABMR) with an R4 score of 0.82, 2 patients showed an R5 phenotype (Fully-developed ABMR), while one patient showed R6 phenotype (Late ABMR) and another one showed a mixed phenotype between R5 (fully-developed ABMR) and R6 (late ABMR). There were no statistically significant differences between both groups regarding R4 phenotype (Early ABMR) or R6 phenotype (Late ABMR). There was no molecular signal for the presence of mixed rejection in AMR-biopsies or any biopsies for which MMDx analysis was available.

**Supplemental Item 10.** In-depth analysis of cases that were reclassified as AMR or probable AMR according to Banff 2022 classification.

After reanalyzing the data according to Banff 2022 classification, one patient has been classified as AMR, who had previously been classified as non-AMR due to the presence of recurrent IgA nephropathy, and another patient was classified as probable AMR, who had previously been classified as non-AMR due to g1ptc0v0 with MMDx AMR score of 0.09 (cutoff > 0.2). In the initial analysis according to Banff 2019, the first patient had g1ptc0C4d0 and minimal mesangial IgA deposition, which is why g1 was not sufficient to allow for a diagnosis of AMR despite the presence of increased AMR transcripts in MMDx. In the Banff 2022 classification, the patient still did not fulfill the MVI criterion (g+ptc ≥2), but due to presence of dnDSA and increased AMR transcripts, a diagnosis of AMR was made. This patient was in the control group and underwent protocol biopsy 14.2 months after baseline. Dd-cfDNA was undulating around the cutoff of 50 copies/mL and one measurement was above the cutoff (57 copies/mL). The second patient, who was classified as probable AMR according to Banff 2022 classification due to DSA+, but MVI score and AMR transcripts below threshold, was in the intervention group and underwent dd-cfDNA guided biopsy at 5.7 months after baseline. Dd-cfDNA increased to 67 copies/mL once before biopsy, but afterwards decreased spontaneously to normal values.

**
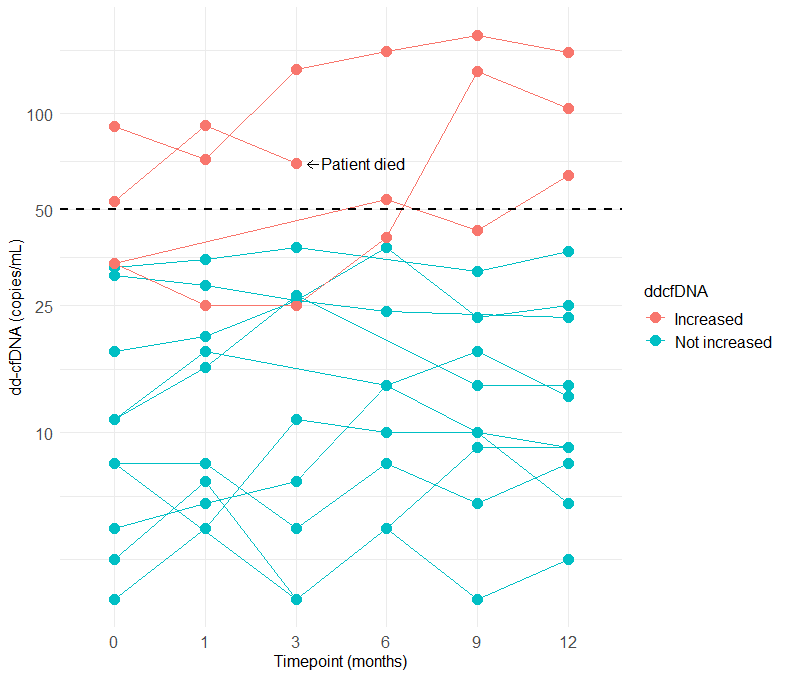
**

**Supplemental Figure 1.** Absolute donor-derived cell-free DNA in 14 patients without kidney biopsy. Four patients had increased dd-cfDNA, one of which died before study completion. The remaining three patients refused protocol biopsy despite increased dd-cfDNA indicating AMR.


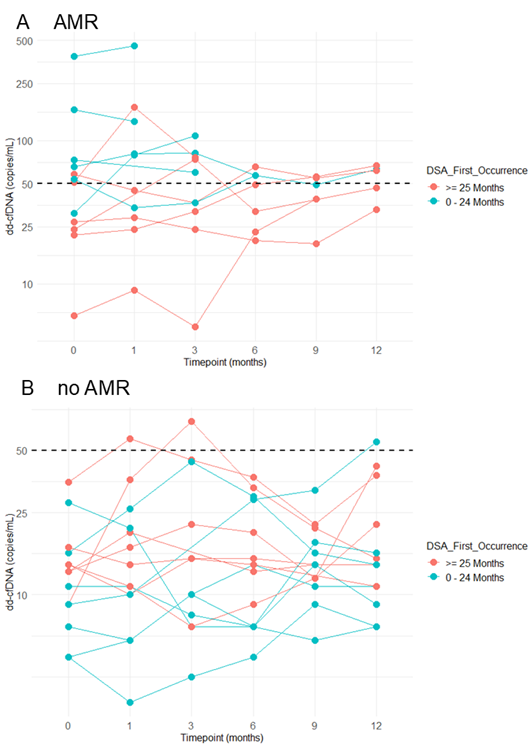


**Supplemental Figure 2.** Absolute donor-derived cell-free DNA in **A)** patients with biopsy-proven AMR, grouped by the time since first DSA occurrence according to the strata used for randomization (0-24 months vs. ≥ 25 months). **B)** patients with non-AMR histopathological diagnosis, grouped by the time since first DSA occurrence according to the strata used for randomization (0-24 months vs. ≥ 25 months)


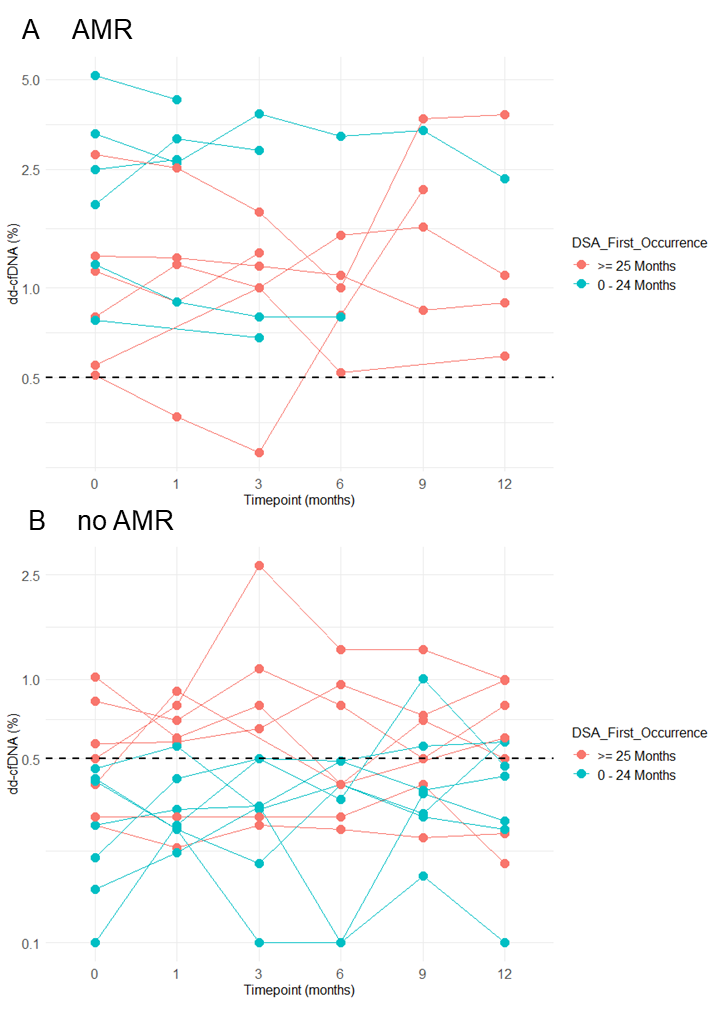


**Supplemental Figure 3.** Relative donor-derived cell-free DNA in **A)** patients with biopsy-proven AMR, grouped by the time since first DSA occurrence according to the strata used for randomization (0-24 months vs. ≥ 25 months). **B)** patients with non-AMR histopathological diagnosis, grouped by the time since first DSA occurrence according to the strata used for randomization (0-24 months vs. ≥ 25 months)


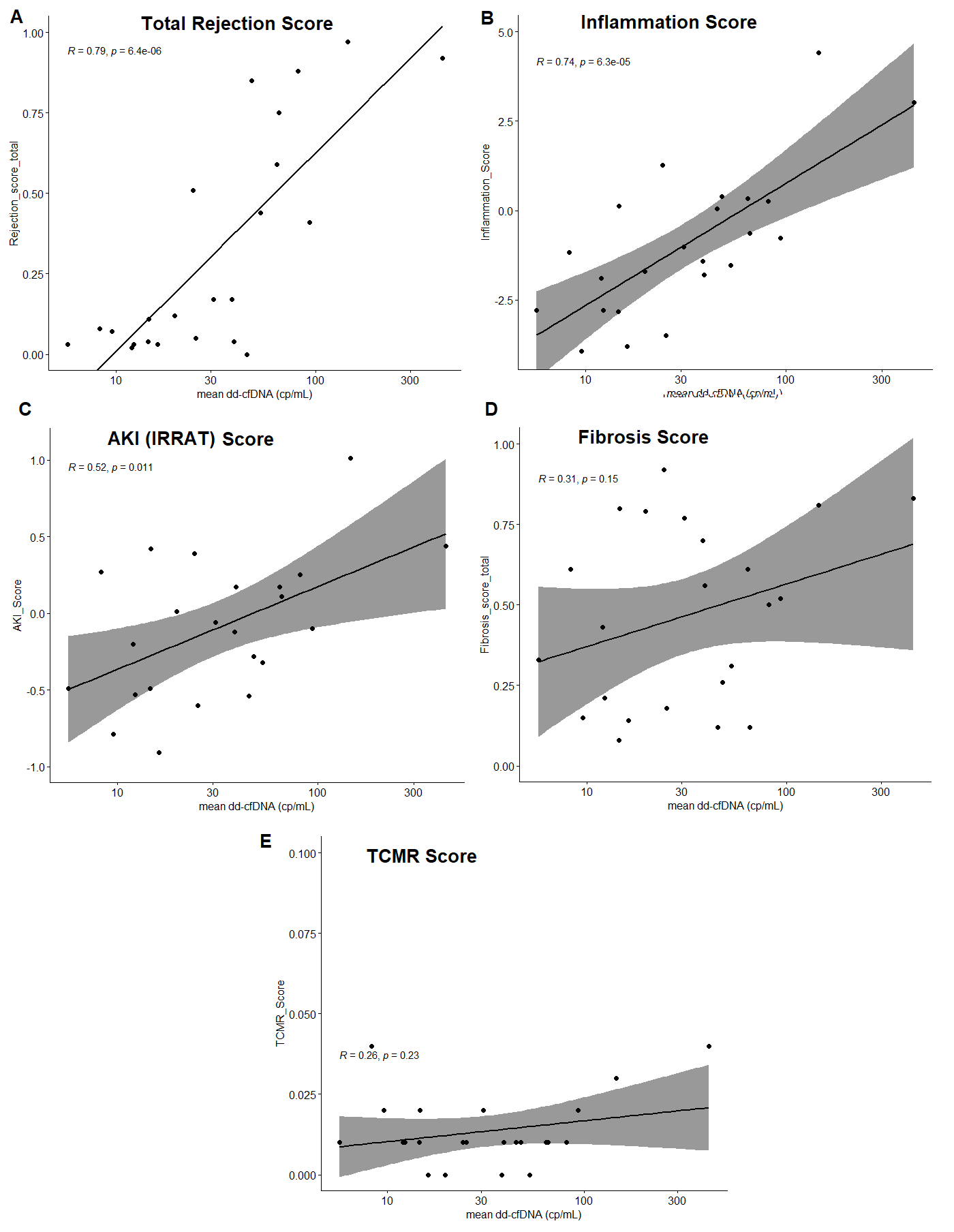


**Supplemental Figure 4.** Pearson correlation of log transformed mean absolute dd-cfDNA with different molecular scores in MMDx: A) total rejection score, B) inflammation score, C) acute kidney injury (AKI) or injury-repair response–associated transcripts (IRRAT) score, D) fibrosis score, E) T cell mediated rejection (TCMR) score.

**Supplemental Table 1.** Follow-up data from patients with biopsy-proven antibody-mediated rejection (AMR) during the study period including treatments and key clinical and laboratory outcomes. IVIG, intravenous immunoglobulins; PLEX, plasma exchange; eGFR, estimated glomerular filtration rate; IQR, interquartile range; uACR, urinary albumin creatinine ratio; MFI, mean fluorescence intensity.

|  | **Intervention group with AMR** | **Control group with AMR** | **P value** |
| --- | --- | --- | --- |
| **N** | 7 | 5 |  |
|  |  |  |  |
| **Treatment** |  |  |  |
| - None | 2 (29%) | - |  |
| - IVIG/PLEX | 1 (14%) | - |  |
| - Placebo in clinical trial | 2 (29%) | - |  |
| - Felzartamab in clinical trial | - | 2 (40%) |  |
| - Belatacept | 1 (14%) | 3 (60%) |  |
| - Blinded study medication | 1 (14%) | - |  |
|  |  |  |  |
| **Outcomes** |  |  |  |
| Overall survival | 7/7 (100%) | 5/5 (100%) | 1.00 |
| Graft survival | 6/7 (86%) | 5/5 (100%) | 1.00 |
| eGFR slope/year at 24 months  median (IQR) | -5.4 (-8.7; -0.1) | -1.8 (-3.2; 1.1) | 0.34 |
| uACR at 24 months | 268 (90; 2238) | 34 (24; 41) | 0.08 |
| MFI at 24 months | 934 (493; 16357) | 10655 (4605; 19698) | 0.64 |
| Severe Infection | 2 (29%) | 2 (40%) | 1.00 |

**Supplemental Table 2.** Contingency table and diagnostic test metrics of maximum intraindividual increase of > 25% compared to the 12 months mean in absolute dd-cfDNA for AMR in DSA+ KTR. Prev, Prevalence of AMR; Acc, accuracy; Sens, sensitivity; Spec, specificity; PPV, positive predictive value; NPV, negative predictive value.

|  | AMR | no AMR | Total |  |
| --- | --- | --- | --- | --- |
| > 25% increase | 6 | 12 | 18 | PPV 0.33 |
| ≤ 25% increase | 6 | 2 | 8 | NPV 0.25 |
| Total | 12 | 14 | 26 | Prev 0.46 |
|  | Sens 0.50 | Spec 0.14 |  | Acc 0.31 |

**Supplemental Table 3.** Contingency table and diagnostic test metrics of a combined criterium of longitudinal absolute dd-cfDNA monitoring with a cutoff of 50 copies/mL or maximum intraindividual increase of > 25% compared to the 12 months mean in absolute dd-cfDNA for AMR in DSA+ KTR. Prev, Prevalence of AMR; Acc, accuracy; Sens, sensitivity; Spec, specificity; PPV, positive predictive value; NPV, negative predictive value.

|  | AMR | no AMR | Total |  |
| --- | --- | --- | --- | --- |
| > 25% increase or > 50 cp/mL | 12 | 12 | 18 | PPV 0.50 |
| ≤ 25% increase  and ≤ 50 cp/mL | 0 | 2 | 8 | NPV 1.00 |
| Total | 12 | 14 | 26 | Prev 0.46 |
|  | Sens 1.00 | Spec 0.14 |  | Acc 0.54 |

**Supplemental Table 4.** Contingency table and diagnostic test metrics of baseline absolute dd-cfDNA with a cutoff of 50 copies/mL for AMR in DSA+ KTR. Prev, Prevalence of AMR; Acc, accuracy; Sens, sensitivity; Spec, specificity; PPV, positive predictive value; NPV, negative predictive value.

|  | AMR | no AMR | Total |  |
| --- | --- | --- | --- | --- |
| > 50 cp/mL | 7 | 0 | 7 | PPV 1.00 |
| ≤ 50 cp/mL | 5 | 14 | 19 | NPV 0.74 |
| Total | 12 | 14 | 26 | Prev 0.46 |
|  | Sens 0.58 | Spec 1.00 |  | Acc 0.81 |

**Supplemental Table 5.** Contingency table and diagnostic test metrics of longitudinal relative dd-cfDNA monitoring with a cutoff of 0.5 % for AMR in DSA+ KTR. Prev, Prevalence of AMR; Acc, accuracy; Sens, sensitivity; Spec, specificity; PPV, positive predictive value; NPV, negative predictive value.

|  | AMR | no AMR | Total |  |
| --- | --- | --- | --- | --- |
| > 0.5% | 12 | 9 | 21 | PPV 0.57 |
| ≤ 0.5% | 0 | 5 | 5 | NPV 1.00 |
| Total | 12 | 14 | 26 | Prev 0.46 |
|  | Sens 1.00 | Spec 0.36 |  | Acc 0.65 |

**Supplemental Table 6.** Contingency table and diagnostic test metrics of longitudinal uACR monitoring with a cutoff of 1000 mg/g for AMR in DSA+ KTR. Prev, Prevalence of AMR; Acc, accuracy; Sens, sensitivity; Spec, specificity; PPV, positive predictive value; NPV, negative predictive value.

|  | AMR | no AMR | Total |  |
| --- | --- | --- | --- | --- |
| > 1000 mg/g | 4 | 1 | 5 | PPV 0.80 |
| ≤ 1000 mg/g | 8 | 13 | 21 | NPV 0.62 |
| Total | 12 | 14 | 26 | Prev 0.46 |
|  | Sens 0.33 | Spec 0.93 |  | Acc 0.65 |

**Supplemental Table 7.** Contingency table and diagnostic test metrics of MFI doubling from baseline to study completion for AMR in DSA+ KTR. Prev, Prevalence of AMR; Acc, accuracy; Sens, sensitivity; Spec, specificity; PPV, positive predictive value; NPV, negative predictive value; MFI, mean fluorescence activity of the immunodominant de novo donor-specfic anti-HLA antibody.

|  | AMR | no AMR | Total |  |
| --- | --- | --- | --- | --- |
| MFI doubling | 0 | 1 | 1 | PPV 0.00 |
| No MFI doubling | 12 | 13 | 25 | NPV 0.52 |
| Total | 12 | 14 | 26 | Prev 0.46 |
|  | Sens 0.00 | Spec 0.93 |  | Acc 0.5 |

**Supplemental Table 8.** Contingency table and diagnostic test metrics of MFI halving from baseline to study completion for AMR in DSA+ KTR. Prev, Prevalence of AMR; Acc, accuracy; Sens, sensitivity; Spec, specificity; PPV, positive predictive value; NPV, negative predictive value; MFI, mean fluorescence activity of the immunodominant de novo donor-specfic anti-HLA antibody.

|  | AMR | no AMR | Total |  |
| --- | --- | --- | --- | --- |
| MFI halving | 2 | 4 | 6 | PPV 0.33 |
| No MFI halving | 10 | 10 | 20 | NPV 0.50 |
| Total | 12 | 14 | 26 | Prev 0.46 |
|  | Sens 0.17 | Spec 0.71 |  | Acc 0.46 |

**Supplemental Table 9.** Contingency table and diagnostic test metrics of longitudinal absolute dd-cfDNA monitoring with a cutoff of 50 copies/mL for AMR in DSA+ KTR according to Banff 2022 classification with probable AMR classified as AMR. Prev, Prevalence of AMR; Acc, accuracy; Sens, sensitivity; Spec, specificity; PPV, positive predictive value; NPV, negative predictive value.

|  | AMR | no AMR | Total |  |
| --- | --- | --- | --- | --- |
| >50 cp/mL | 12 | 1 | 13 | PPV 0.92 |
| ≤50 cp/mL | 2 | 11 | 13 | NPV 0.85 |
| Total | 12 | 14 | 26 | Prev 0.46 |
|  | Sens 0.86 | Spec 0.92 |  | Acc 0.88 |

**Supplemental References**

**1** Tait BD, Süsal C, Gebel HM, Nickerson PW, Zachary AA, Claas FH, Reed EF, Bray RA, Campbell P, Chapman JR, Coates PT, Colvin RB, Cozzi E, Doxiadis II, Fuggle SV, Gill J, Glotz D, Lachmann N, Mohanakumar T, Suciu-Foca N, Sumitran-Holgersson S, Tanabe K, Taylor CJ, Tyan DB, Webster A, Zeevi A, Opelz G. Consensus guidelines on the testing and clinical management issues associated with HLA and non-HLA antibodies in transplantation. Transplantation. 2013 Jan 15;95(1):19-47. doi: 10.1097/TP.0b013e31827a19cc.

**2** Oellerich M, Shipkova M, Asendorf T, et al. Absolute quantification of donor-derived cell-free DNA as a marker of rejection and graft injury in kidney transplantation: results from a prospective observational study. Am J Transplant. 2019;19:3087–3099.

**3** Inker LA, Eneanya ND, Coresh J, Tighiouart H, Wang D, Sang Y, Crews DC, Doria A, Estrella MM, Froissart M, Grams ME, Greene T, Grubb A, Gudnason V, Gutiérrez OM, Kalil R, Karger AB, Mauer M, Navis G, Nelson RG, Poggio ED, Rodby R, Rossing P, Rule AD, Selvin E, Seegmiller JC, Shlipak MG, Torres VE, Yang W, Ballew SH, Couture SJ, Powe NR, Levey AS; Chronic Kidney Disease Epidemiology Collaboration. New Creatinine- and Cystatin C-Based Equations to Estimate GFR without Race. N Engl J Med. 2021 Nov 4;385(19):1737-1749. doi: 10.1056/NEJMoa2102953. Epub 2021 Sep 23. PMID: 34554658; PMCID: PMC8822996.

**4** Lachmann N, Niemann M, Reinke P, Budde K, Schmidt D, Halleck F, Pruß A, Schönemann C, Spierings E, Staeck O. Donor-Recipient Matching Based on Predicted Indirectly Recognizable HLA Epitopes Independently Predicts the Incidence of De Novo Donor-Specific HLA Antibodies Following Renal Transplantation. Am J Transplant. 2017 Dec;17(12):3076-3086. doi: 10.1111/ajt.14393. Epub 2017 Jul 28. PMID: 28613392.

**5** Schinstock CA, Cosio F, Cheungpasitporn W, Dadhania DM, Everly MJ, Samaniego-Picota MD, Cornell L, Stegall MD. The Value of Protocol Biopsies to Identify Patients With De Novo Donor-Specific Antibody at High Risk for Allograft Loss. Am J Transplant. 2017 Jun;17(6):1574-1584. doi: 10.1111/ajt.14161. Epub 2017 Jan 25. PMID: 27977905; PMCID: PMC5445019.

**6** Huang E, Sethi S, Peng A, Najjar R, Mirocha J, Haas M, Vo A, Jordan SC. Early clinical experience using donor-derived cell-free DNA to detect rejection in kidney transplant recipients. Am J Transplant. 2019 Jun;19(6):16^6^3-1670. doi: 10.1111/ajt.15289. Epub 2019 Mar 29. PMID: 30725531.

**7** Jordan SC, Bunnapradist S, Bromberg JS, Langone AJ, Hiller D, Yee JP, Sninsky JJ, Woodward RN, Matas AJ. Donor-derived Cell-free DNA Identifies Antibody-mediated Rejection in Donor Specific Antibody Positive Kidney Transplant Recipients. Transplant Direct. 2018 Aug 20;4(9):e379. doi: 10.1097/TXD.0000000000000821
